# Extracranial vertebral artery stenosis patients that may benefit from stent placement: post-hoc analysis from randomized controlled trials

**DOI:** 10.3389/fneur.2025.1719750

**Published:** 2026-02-12

**Authors:** Adnan I. Qureshi, Nived J. Ranjini, Yilun Huang, Hatem Tolba, Mojgan Golzy, Pashmeen Lakhani, Akash Roy, Christy N. Cassarly, Renee’ H. Martin, William J. Powers

**Affiliations:** 1 Zeenat Qureshi Stroke Institute, Columbia, MO, United States; 2 University of Missouri, Columbia, MO, United States; 3 Department of Public Health Sciences, Medical University of South Carolina, Charleston, SC, United States; 4 Department of Neurology, Duke University School of Medicine, Durham, NC, United States

**Keywords:** extracranial vertebral artery stenosis, randomized controlled trial, stent placement, stroke, vertebrobasilar ischemic stroke

## Abstract

**Background:**

Stent placement is a treatment option intended to reduce the risk of ischemic events in patients with extracranial vertebral artery stenosis (EVAS). However, the patient subgroups that can potentially benefit from stent placement are not well defined.

**Methods:**

We performed an exploratory pooled analysis of patients with EVAS (≥50% in severity) enrolled within 30 days of the qualifying event of ischemic stroke from two randomized controlled trials evaluating stent placement and medical treatment alone. We compared the risk of vertebrobasilar arterial distribution ischemic stroke and/or death (and other endpoints) post-randomization between the two groups using adjusted Cox proportional hazards models and estimated the event-free survival during 2-year post-randomization follow-up using Kaplan–Meier curves.

**Results:**

A total of 94 patients (50 randomized to stent placement and 44 assigned to best medical treatment only) were included in the analysis. After adjusting for age and use of antihypertensive medication at baseline, the risks of vertebrobasilar arterial distribution ischemic stroke and/or death (hazard ratio [HR]: 0.4, 95% confidence interval [CI]: 0.1–1.1, *p* = 0.08), any stroke (HR: 0.2, 95% CI: 0.1–0.8, *p* = 0.02), ischemic stroke (HR 0.1, 95% CI: 0.03, 0.6, *p* = 0.01), and any stroke and/or death (HR 0.3, 95% 0.1–0.9, *p* = 0.03) were found to be lower in patients who underwent stent placement. The estimated proportion of patients with vertebrobasilar arterial distribution ischemic stroke-free survival at 2-year post-randomization was higher in patients randomized to stent placement (92% vs. 80.9%, log-rank *p*-value = 0.06).

**Conclusion:**

Patients with EVAS can potentially benefit from stent placement within 30 days following an ischemic stroke and should be further evaluated in clinical trials.

## Introduction

Extracranial vertebral artery stenosis (EVAS) is relatively common among patients with ischemic events in the vertebral and basilar arterial distributions (1–4). Of the 407 patients in the New England Medical Center Posterior Circulation Registry, 80 (20%) of them with ischemic events in the vertebrobasilar arterial distribution had either extracranial vertebral artery occlusion or high-grade stenosis (2). If we consider that there are 600,000 ischemic strokes and 240,000 transient ischemic attacks (TIAs) in the United States every year (5, 6), we can estimate that 20% (*n* = 168,000) of these events are likely to be located in the vertebrobasilar arterial distribution (7). Approximately 15% of ischemic strokes and TIAs located in the vertebrobasilar arterial distribution are related to EVAS (2, 8). Therefore, approximately 25,200 patients every year have ischemic stroke and TIAs in the United States that are related to EVAS. Despite the use of antiplatelet medications, recurrent ischemic strokes remain a relatively common occurrence in approximately 16% of these patients within 3 months following the first ischemic event (9–12). Stent placement is a treatment option aimed at reducing the risk of ischemic stroke in patients with high-grade EVAS, although the therapeutic efficacy has not yet been proven (13). In patients with TIAs or ischemic stroke related to EVAS who have symptoms despite optimal medical treatment, the recommendation from the 2021 (American Heart Association/American Stroke Association) guidelines for Prevention of Stroke in Patients with Stroke and TIA specifies that “the usefulness of stenting is not well established.” (14) This recommendation was based on the inconclusive results of three small randomized controlled trials (RCTs), which did not demonstrate any benefit of stent placement over best medical treatment in patients with EVAS. In one of the trials, Carotid And Vertebral Artery Transluminal Angioplasty Study (CAVATAS), only eight patients were treated with either angioplasty (*n* = 6) or stent placement (*n* = 2) (15–17). However, the broad confidence intervals indicate that a larger trial focusing on high-risk patients is required to conclusively evaluate the therapeutic benefit of stent placement in patients with EVAS (14).

We performed this subgroup analysis of the two most recent RCTs to further study the relative benefit on various endpoints of stent placement (compared with medical treatment) in high-risk patients with EVAS.

## Methods

Data from patients with EVAS from two RCTs, the Vertebral Artery Stenting Trial (VAST) (15) and the Vertebral Artery Ischemia Stenting Trial (VIST), were analyzed (16). Public access files available at the University of Cambridge repository were utilized for our analysis (18).

Both VAST (15, 19) and VIST (16) recruited patients with a qualifying ischemic event (vertebrobasilar TIA or minor ischemic stroke) within the past 6 months. These patients had intracranial or extracranial vertebral artery stenosis of 50% or greater, confirmed using at least two imaging modalities in the VAST. For the VIST, confirmation was made using magnetic resonance angiography (MRA), preferably contrast-enhanced, contrast-enhanced computed tomography angiography (CTA), or digital subtraction angiography. A total of 115 patients from 7 centers were recruited for the VAST (15, 19), and 179 patients from 14 centers were recruited for the VIST (16). In the VIST (16), recruitment was limited to patients with a qualifying ischemic event within 3 months after the first 100 patients were enrolled, as interim data showed that stroke risk was highest in the first 3 months. The patients were randomly assigned to receive either stent placement with best medical treatment or best medical treatment alone in a 1:1 ratio in both VAST (15, 19) and VIST (16). The choice of stent was at the discretion of the interventionalist performing the procedure; although in the VIST, the stent used required a Conformité Européenne mark approval. The patients were treated predominantly using balloon expandable stents, which were used in 72 and 92% of the patients treated in VAST and VIST, respectively. Primary angioplasty was performed in 2 and 5% of the patients treated in VAST and VIST, respectively. In VIST and VAST, patients allocated to the stent group were required to receive clopidogrel 75 mg daily in addition to aspirin (or a vitamin K antagonist in VAST) for at least 30 days post-procedure.

### Follow-up and outcome assessments

In the VAST (15, 19), follow-up visits were planned at day 1 and then at 1, 6, and 12 months after stent placement (or randomization in the non-stent treatment group) and every year thereafter. In the VIST (16), follow-up visits occurred at day 1 and at 1 and 12 months after stent placement (or randomization in the non-stent treatment group). A follow-up angiogram was not a requirement in these studies. Both VIST and VAST recommended a non-invasive test, a duplex ultrasound or MR/CT angiography during the 12-month follow up visit. In addition, telephone follow-ups were conducted at 6 months, 2 years, and annually thereafter by the coordinating center, overseen by a designated stroke physician or neurologist. The median follow-up time for the VAST and VIST was 1,096 days (interquartile range (IQR): 475–1,498 days) and 1,278 days (IQR: 767–1,717 days), respectively. The occurrence of any stroke, including stroke in the vertebrobasilar arterial distribution, and death from any cause during follow-up was ascertained in both trials. Stroke was defined based on the occurrence of a new focal neurological deficit of presumed vascular origin that lasts longer than 24 h, regardless of its severity. Stroke was further classified into ischemic stroke and intracerebral hemorrhage. The identification and classification of stroke were performed by site investigators.

### Statistical analysis

A high-risk group of patients with EVAS was analyzed from the overall cohort, selected based on whether the qualifying ischemic event was ischemic stroke and whether the time interval between the qualifying ischemic event and randomization was ≤ 30 days. Previous studies have shown that patients presenting with ischemic stroke have a higher risk of recurrent ischemic stroke compared to those presenting with TIA (20, 21). We used the cutoff of ≤ 30 days based on two studies: one conducted by Gulli et al. (11, 22) and the Vertebrobasilar Flow Evaluation and Risk of Transient Ischemic Attack and Stroke Trial (VERiTAS) (23). These studies found that the majority of recurrent ischemic events occur within 30 days after the first ischemic event in patients with EVAS and vertebrobasilar stenosis, respectively. The cutoff of ≤ 30 days between a qualifying ischemic event and enrollment has been used in other studies, such as the Stenting versus Aggressive Medical Therapy for Intracranial Arterial Stenosis (SAMMPRIS) trial (24).

The primary endpoint was vertebrobasilar arterial distribution ischemic stroke and/or death during follow-up. Other exploratory endpoints were analyzed, including any stroke, ischemic stroke, vertebrobasilar artery distribution ischemic stroke, any stroke or death, or a composite of any stroke and/or death ≤ 1-month post-randomization and vertebrobasilar arterial distribution ischemic stroke.

In the designated high-risk subgroup of patients who were randomized within 30 days following a qualifying event of ischemic stroke, continuous and categorical characteristics are presented as means with standard deviations and frequencies, respectively. We employed analysis of variance and chi-squared tests to compare continuous and categorical variables between patients randomized to receive a stent and those receiving the best medical management, respectively. We performed Cox proportional hazards models to estimate the effect of stent placement on the endpoints. We adjusted for age and baseline factors that were statistically different at a *p*-value of < 0.05 in the univariate analysis. We tested the validity of the proportional hazards for all endpoints assumptions using Cox.zph, which yielded a non-significant result (*p* > 0.05), indicating no violation of the assumptions. For the three selected endpoints of vertebrobasilar arterial distribution ischemic stroke-free survival, any stroke-free survival, and any stroke ≤ 1-month post-randomization and vertebrobasilar arterial distribution ischemic stroke-free survival, we estimated the 2-year event-free rates in patients randomized to stent placement and those randomized to best medical treatment using Kaplan–Meier analysis and compared the survival curves out to 2 years with the log-rank statistical test.

We also estimated adjusted hazard ratios for the primary endpoint of vertebrobasilar arterial distribution ischemic stroke-free survival in other strata defined by the type of qualifying ischemic events and the time interval between qualifying ischemic events and enrollment to validate the assumptions of the high-risk group (see Supplementary materials). In these exploratory analyses, we present uncorrected *p*-values for increased type 1 error due to multiple comparisons. These results should be interpreted as generating hypothesis rather than definitive conclusions.

## Results

The VIST and VAST trials collectively randomized a total of 294 patients, with 244 patients (83.0%) diagnosed with EVAS. Our primary analysis included 94 patients in whom the qualifying ischemic event was ischemic stroke and who were randomized within 30 days following the qualifying ischemic event; 50 and 44 patients randomly underwent stent placement and best medical treatment, respectively. The median follow-up time was 1,111 (IQR 476–1,523) days. There were no significant differences at the *p* < 0.05 level in the baseline demographics and characteristics between the two groups, except for the percentage of patients using antihypertensive drugs at baseline, which was higher in those randomized to stent placement [*p* = 0.048, see Table 1]. This factor was adjusted for along with age in the Cox proportional hazards models.

**Table 1:** Baseline demographic and clinical characteristics of subjects according to the allocated treatment.

Baseline characteristics	Randomized to best medical treatment (*N* = 44)	Randomized to stent placement (*N* = 50)	*P*-value
Age (years), mean	62.9	63.0	0.59
Men, *n* (%)	36 (81.8)	43 (86.0)	0.79
Hypertension at baseline, *n* (%)	27 (61.4)	38 (76.0)	0.19
Hyperlipidemia at baseline	38 (86.4)	42 (84.0)	1
Diabetes mellitus at baseline, *n* (%)	9 (20.5)	9 (18)	0.97
Smoking status at baseline, *n* (%)	13 (29.5)	13 (26)	0.3
History of coronary heart disease, *n* (%)	4 (9.1)	5 (10.0)	1
History of peripheral artery disease, *n* (%)	2 (4.5)	2 (4)	1
History of atrial fibrillation	6 (13.6)	6 (12)	1
Aspirin taken at baseline, *n* (%)	30 (68.2)	40 (80.0)	0.28
Dipyridamole taken at baseline, *n* (%)	10 (22.7)	17 (34.0)	0.33
Clopidogrel taken at baseline, *n* (%)	26 (59.1)	23 (46.0)	0.29
Oral anticoagulants taken at baseline, *n* (%)	4 (9.1)	1 (2.0)	0.29
Antihypertensive drugs use at baseline, *n* (%)^*^	25 (56.8)	39 (78.0)	0.048
Statins/cholesterol-lowering drugs taken at baseline, *n* (%)	40 (90.9)	42 (84.0)	0.49

* *p* < 0.05.

Overall, 14 patients (14.9%) of the 94 experienced vertebrobasilar arterial distribution ischemic stroke and/or death: 9 out of 44 patients (20.5%) in the best medical treatment only group and 5 out of 50 patients (10.0%) in the stent group. The adjusted hazard ratio (HR) for patients randomized to stent placement, after adjusting for age and the use of antihypertensive drugs at baseline, was 0.4, with a 95% confidence interval (CI) of 0.1–1.1. (*p* = 0.08). [Table 2] Regarding other exploratory endpoints, the risk of any stroke (HR 0.2, 95% CI 0.1–0.8, *p* = 0.02), ischemic stroke (HR 0.1, 95% CI 0.03–0.6, *p* = 0.01), and the combination of any stroke and/or death (HR 0.3, 95% CI 0.1–0.9, *p* = 0.01) was lower in patients randomized to stent placement.

**Table 2:** Post-randomization outcomes in subjects according to treatment allocation.

Outcomes	Randomized to best medical treatment^*^ (*N* = 44)	Randomized to stent placement^*^ (*N* = 50)	Hazard ratio (95% confidence interval) ^**^
Vertebrobasilar arterial distribution ischemic stroke or death, *n* (%) ^***^	9 (20.5)	5 (10.0)	0.4 (0.1–1.1), *p* = 0.08
Any stroke or death ≤ 30 days of randomization, *n* (%)	2 (4.5)	1 (2.0)	0.5 (0.04–7.2), *p* = 0.65
Any stroke, *n* (%)	9 (20.5)	3 (6.0)	0.2 (0.1–0.8), *p* = 0.02
Ischemic stroke, *n* (%)	9 (20.5)	2 (4.0)	0.1 (0.03–0.6), *p* = 0.01
Vertebrobasilar arterial distribution ischemic stroke, *n* (%)	5 (11.4)	2 (4.0)	0.3 (0.1–1.7), *p* = 0.17
Intracerebral hemorrhage, *n* (%)	0	1 (2.0)	NA
Transient ischemic attack, *n* (%)	3 (6.8)	3 (6.0)	0.8 (0.2–4.0), *p* = 0.78
Death, *n* (%)	5 (11.4)	3 (6.0)	0.5 (0.1–2.1), *p* = 0.35
Any stroke or death, *n* (%)	12 (27.3)	6 (12.0)	0.3 (0.1–0.9), *p* = 0.03
Patients with any stroke or death ≤ 30 days and vertebrobasilar arterial distribution ischemic stroke after 30 days, *n* (%)	9 (20.5)	6 (12.0)	0.4 (0.2–1.3), *p* = 0.12

* Cumulative events for the total duration of the trials.

** Cox PH model after adjusting for age and antihypertensive drugs used at baseline, for the total duration of the trials.

*** Primary outcome.

**Figure 1:**
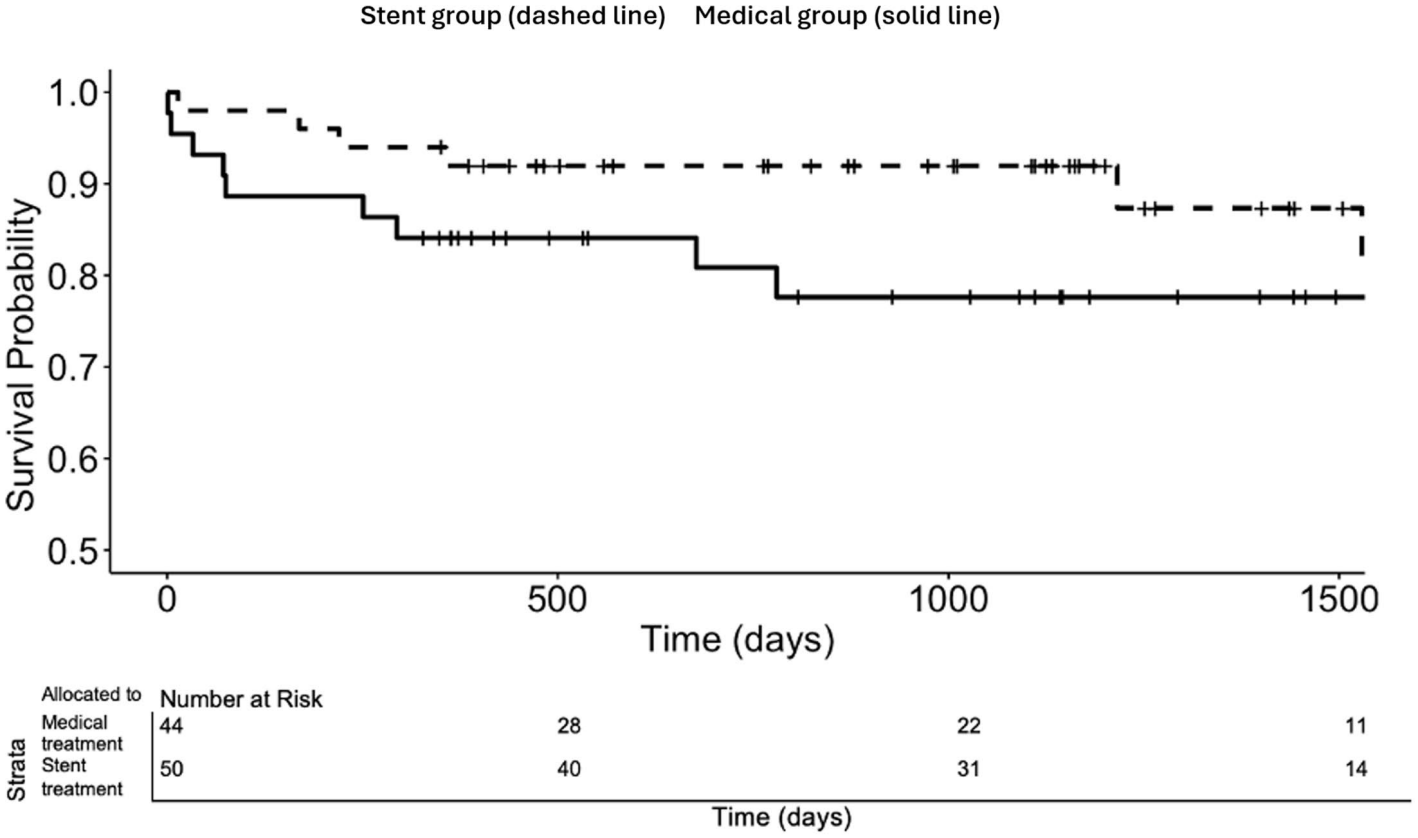
Vertebrobasilar arterial distribution ischemic stroke-free survival according to treatment allocation in subjects randomized ≤ 30 days and qualifying event of ischemic stroke for the total duration of the trials.

**Figure 2:**
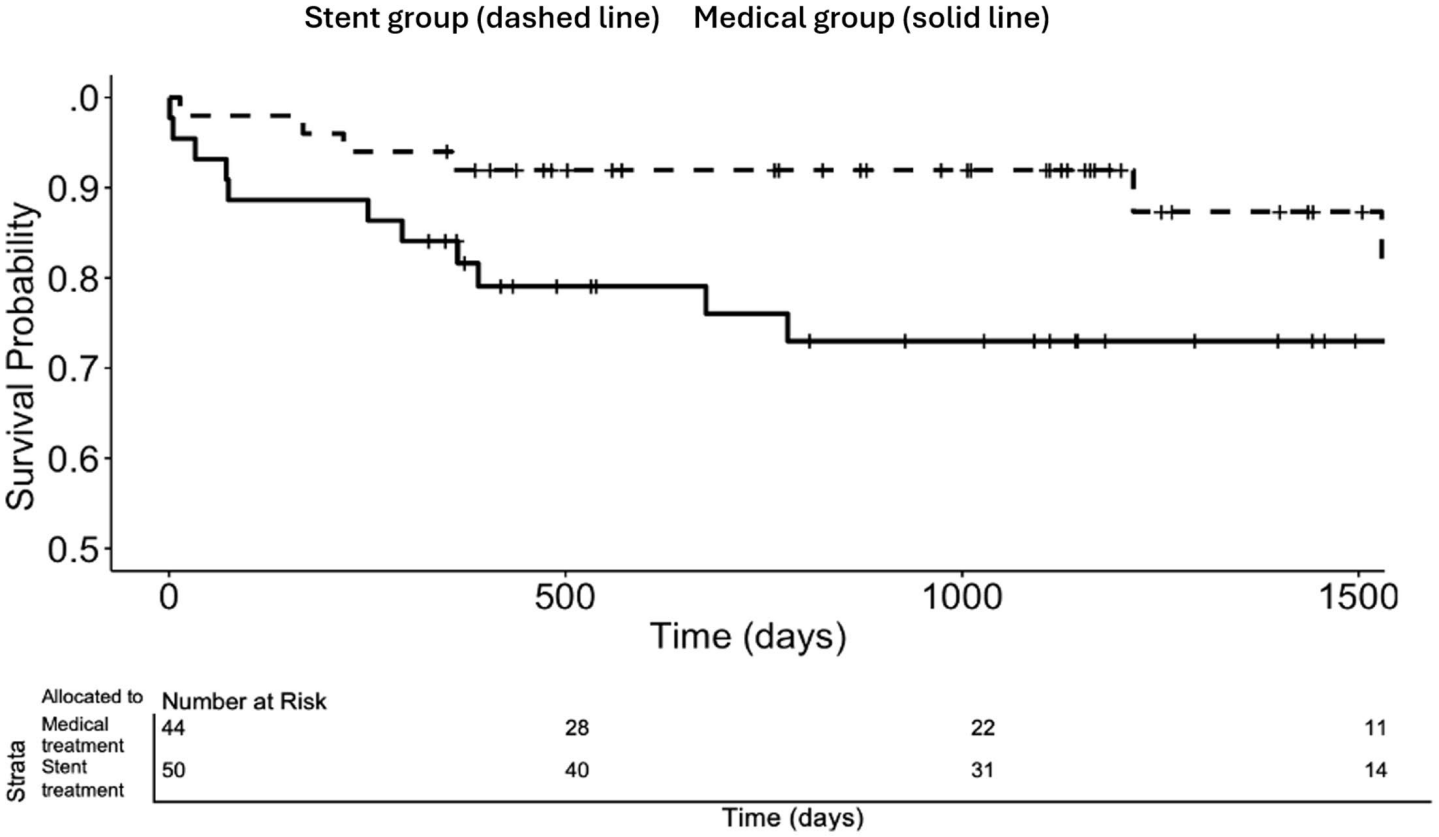
Any stroke-free survival according to treatment allocation in subjects randomized ≤ 30 days and qualifying event of ischemic stroke for the total duration of the trials.

The Kaplan–Meier event-free survival curves are presented in Figures 1 and 2. The estimated proportion of patients with primary endpoint vertebrobasilar arterial distribution ischemic stroke-free survival at 2 years post-randomization was numerically higher with stent placement in subjects randomized ≤ 30 days of an ischemic stroke (92.0% vs. 80.9%, log-rank *p*-value 0.06 at 2 years) [Figure 1]. Furthermore, the proportion of patients with any stroke-free survival at 2 years post-randomization in subjects randomized ≤ 30 days of ischemic stroke was higher with stent placement (92.0% vs. 76.0%, log-rank *p*-value 0.02 at 2 years) [Figure 2].

Compared to the adjusted hazard ratio in the designated high-risk subgroup with a qualifying event of ischemic stroke and enrollment within 30 days, all other subgroup strata based on the type of qualifying event and the time from the qualifying event to randomization showed a higher probability of obtaining test results with differences in the primary outcome that were at least as large as those observed, assuming that the null hypothesis is true (all log-rank *p* > 0.08, Supplementary Figures 1–8).

## Discussion

Stent placement was found to be associated with a reduced rate of vertebrobasilar arterial distribution ischemic stroke and/or death in a group of patients with EVAS. There was an absolute reduction in the composite outcomes by 11–16% at 2 years post-randomization associated with stent placement (compared with best medical treatment) in patients randomized within 30 days following the qualifying event of ischemic stroke. RCTs evaluating the therapeutic benefit of stent placement in patients with EVAS are less common than those evaluating carotid artery stent placement. This is primarily due to the lower prevalence of moderate-to-severe EVAS (25, 26), the absence of revascularization procedures such as endarterectomy performed over several decades, the lack of stents specifically designed for EVAS (27, 28), and limitations in accurately diagnosing stenosis using non-invasive imaging due to the poor visualization of the extracranial segments, which is attributed to the small caliber and tortuosity of the extracranial vertebral arteries (15–17, 29). Therefore, the post-hoc analysis provides a unique opportunity to inform future RCTs evaluating the therapeutic benefit of stents in patients with EVAS.

The potential 30-day time window for performing extracranial vertebral artery stent placement to maximize the therapeutic benefit is consistent with the findings from previous cohort studies (9–11). An analysis of 43 studies representing 36 independent cohorts (12,196 patients) (9) reported that the highest relative risk of recurrent stroke in patients with vertebrobasilar events was seen in studies that enrolled patients in the acute phase (up to 7 days) after the presenting ischemic event. In contrast, the relative risk was markedly lower in studies that recruited patients after the acute phase. Two cohort studies, conducted by Marquardt et al. (10) and Gulli et al. (11), noted that the majority of the recurrent stroke events occurred within the first 30 days in patients with EVAS. Gulli et al. (11) reported a 33% risk for recurrent vertebrobasilar arterial distribution ischemic stroke in the first month in patients with (intra- or extracranial) vertebrobasilar arterial stenosis. Marquardt et al. (10) highlighted the risk of early recurrent ischemic stroke in patients with vertebrobasilar arterial stenosis and cautioned that recurrent stroke may occur in half of the patients prior to medical consultation in the absence of expedient triage. In the VERiTAS trial (23), 42 out of 72 patients with centrally adjudicated atherosclerotic (intra- or extracranial) vertebrobasilar disease ≥ 50% with vertebrobasilar distribution TIAs or stroke within 60 days, reported a previous vertebrobasilar distribution ischemic event, with over half of these events occurring within 30 days of the qualifying ischemic event. A combined analysis of 37 patients with EVAS (22), of which pooled data from Marquardt et al. (10) and Gulli et al. (11), reported that 16% of the patients suffered recurrent vertebrobasilar arterial distribution ischemic stroke within the first month after the index event, with no patients experiencing recurrent ischemic stroke between 30 and 90 days. Studies that did not limit recruitment of patients within a 30-day time window following the first ischemic event have reported a much lower risk of recurrent vertebrobasilar arterial distribution ischemic stroke. For instance, one study reported a recurrence rate of 7.3% among 55 medically treated patients with EVAS within 90 days (30, 31).

The risk of recurrent ischemic stroke or death being higher among patients with ischemic stroke compared with those with TIAs observed in our analysis is supported by previous studies (32). Several studies have demonstrated a higher risk of recurrent ischemic stroke in patients presenting with ischemic stroke compared with those with TIAs (18, 21). The identification of TIAs related to EVAS is challenging since over half of the patients present with transient non-focal neurological symptoms (33). Non-focal symptoms occurred more frequently in patients with a symptomatic EVAS compared to those with carotid artery stenosis, posing a challenge in differentiating TIAs from transient non-ischemic events that have a much lower risk of subsequent ischemic events (33). In an analysis of 863 non-cardioembolic posterior circulation ischemic stroke patients from the Chinese IntraCranial AtheroSclerosis Study that presented with minor stroke (34), it was found that the presence of multiple acute cerebral infarcts (HR - 1.6, 95% CI 1.0–2.6) is an independent risk factor for recurrent ischemic stroke or TIAs within 1 year. Karameshev et al. (35) also reported that the benefit of stent placement was higher when ischemic stroke was the qualifying ischemic event (odds ratio [OR] 0.5) compared to when TIAs were the qualifying event (OR 2.1). This was based on an observational study of 39 patients with EVAS (70–99% in severity) who were treated either medically or with stent placement and followed for a mean period of 2.8 years.

The potential patients who may be eligible based on the criteria need to be considered. The VAST recruited 30 patients who met the high-risk criteria over 60 months at 7 centers (19). The VIST recruited 64 patients who met the high-risk criteria over 88 months at 14 centers (16). The exact duration for which each site screened and recruited patients in the study was not recorded (16). Data from a prospective registry called Angiographically Confirmed Vertebral Artery Disease (ACVAD) were analyzed. This registry collected data on all adult patients who underwent cerebral angiography at a single institution and were diagnosed with either symptomatic or asymptomatic extra- or intracranial vertebral artery stenosis ≥ 50% or occlusion, over a period of 17 months (36). A total of 10 patients met the criteria (see Supplementary material for flow diagram, in line with the Consolidated Standards of Reporting Trials (CONSORT) guidelines). A follow-up study (ACVAD 2) at the same institution evaluated all patients with ischemic stroke related to large vessel atherosclerosis admitted over a period of 2 years. A total of 27 out of 75 acute ischemic stroke patients met the criteria of: 1/non-disabling vertebrobasilar artery distribution ischemic stroke that occurred within 1 month and EVAS classified as moderate to severe on CTA or MRA (see Supplementary material for flow diagram, in line with the CONSORT guidelines).

A limitation of this analysis is that information regarding the treatment between qualifying events and randomization, and their relationship to the recurrence of vertebrobasilar arterial distribution ischemic stroke, was not collected in either study. The determination of stenosis severity was based on non-invasive tests in both trials, which may have resulted in the inclusion of patients with a severity of < 50% due to potential overestimation by these non-invasive tests. Furthermore, 4 out of 57 patients in the VIST and 23 out of 91 patients in the VAST had EVAS classified as < 50% in severity on angiography performed as part of the stent procedure. The rate of overestimation of severity in both trials appeared higher than that reported in a systematic review of 11 studies (37). The sensitivity of CTA (single study) and the pooled sensitivities of contrast-enhanced MRA and color Duplex were 100, 93.9, and 70.2%, respectively, for identifying EVAS (50–99% in severity). The specificities for CTA, MRA, and color Duplex were 95.2, 94.8, and 97.7%. However, specificities for MRA and color duplex demonstrated substantial heterogeneity. The measurement of the severity of EVAS is not standardized, and the North American Symptomatic Carotid Endarterectomy Trial criteria for measuring the severity of carotid artery stenosis were utilized in the majority of studies (38). The effect of contralateral vertebral artery hypoplasia or occlusion, which may augment the incidence of vertebrobasilar arterial distribution ischemic stroke in patients with EVAS, was not evaluated in either study (39, 40). Furthermore, quantitative MRA was not utilized to assess the flow characteristics for patient stratification (41, 42). The endpoint of the study was defined as vertebrobasilar arterial distribution ischemic stroke and/or death. Neither of these study endpoints was adjudicated by an independent committee. Death could be related to any cause and may not be related to recurrent vertebrobasilar arterial distribution ischemic stroke. It was not necessary for the trials to differentiate between deaths related to vertebrobasilar arterial distribution ischemic stroke and those unrelated to it. Drug-eluting stents (DESs) were not used in these trials, which may reduce the risk of restenosis in patients with EVAS (43). In a systematic review of 27 studies (13), DESs were associated with lower restenosis rates (11.0% vs. 30.0%) at a mean follow-up duration of 24 months. A catheter-based angiogram was not required as part of follow-up imaging due to the risk associated with angiography. Therefore, we are unable to comment upon the rate of restenosis at 6–12 months post-stent placement. In another study, the rate of recurrent symptoms of vertebrobasilar arterial distribution ischemic stroke (2.8% vs. 11.3%) was lower in patients treated with DESs compared to those treated with bare metal stents (27). Intensive medical treatment, such as the one used in the SAMMPRIS trial, which included aggressive medical management characterized by the use of dual antiplatelet therapy for 90 days, high-dose statin therapy, and other risk factors and lifestyle modification, was not used in either of the trials, which could have modified the risk (34).

Despite the limitations, our evidence suggests that patients with EVAS (50% or greater in severity) with ischemic stroke as the qualifying event who can be randomized within 30 days following a qualifying ischemic event may potentially benefit from stent placement and should be further evaluated in clinical trials.

## Data Availability

Publicly available datasets were analyzed in this study. These data can be found here: Markus HS, Harshfield EL, Compter A, et al. Research data supporting “Stenting for symptomatic vertebral artery stenosis: a preplanned pooled individual patient data analysis.” Published online 16 May 2019. doi: 10.17863/CAM.39611.
