# Supplementary material for: Extracranial vertebral artery stenosis patients that may benefit from stent placement: post-hoc analysis from randomized controlled trials
Source: Front Neurol. 2026 Feb 12;16:1719750. doi: 10.3389/fneur.2025.1719750 (PMC12937133; doi:10.3389/fneur.2025.1719750)
Supplement: Supplementary file 1 [file Supplementary_file_1.docx]

**SUPPLEMENTARY FIGURES**

Supplementary Figure 1: Vertebrobasilar arterial distribution ischemic stroke free survival for patients with ischemic stroke as the qualifying event who were randomized > 30 days. (N=70) (adjusted HR: 0.779 95% CI: 0.174 - 3.481, p=0.743)

Stent group (dashed line) Medical group (solid line)


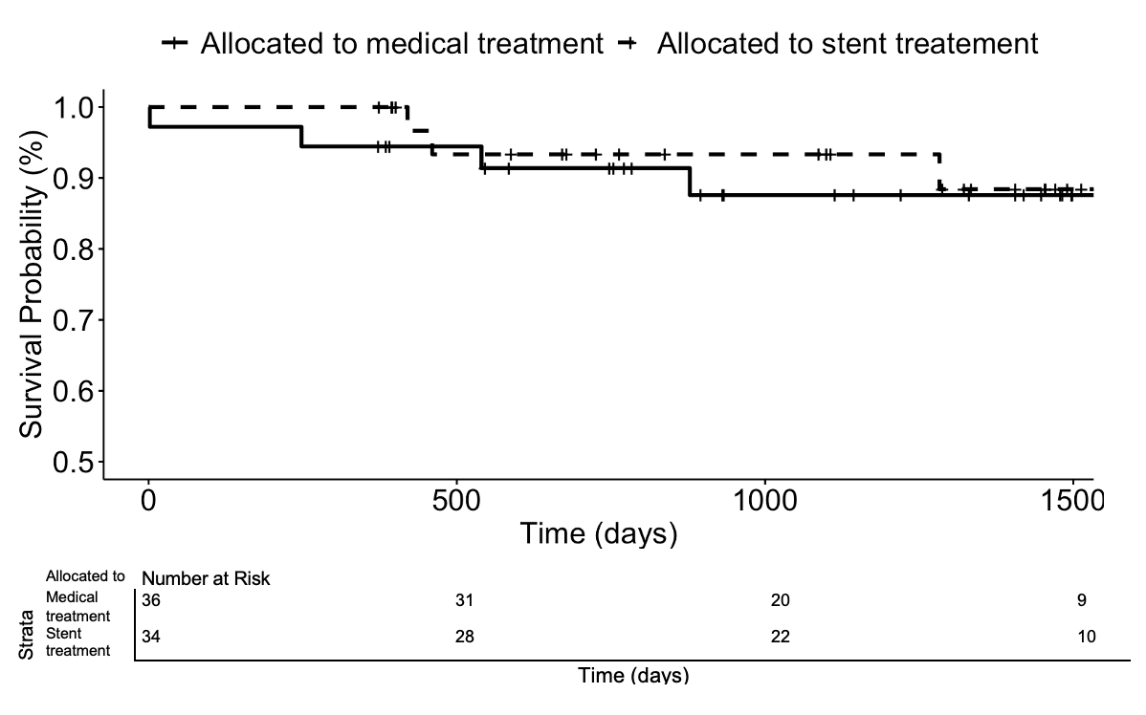


Supplementary Figure 2: Vertebrobasilar arterial distribution ischemic stroke free survival for patients with ischemic stroke as the qualifying event who were randomized within any number of days. (N=164) (adjusted HR: 0.548, 95% CI: 0.227–1.323, p=0.181)

Stent group (dashed line) Medical group (solid line)


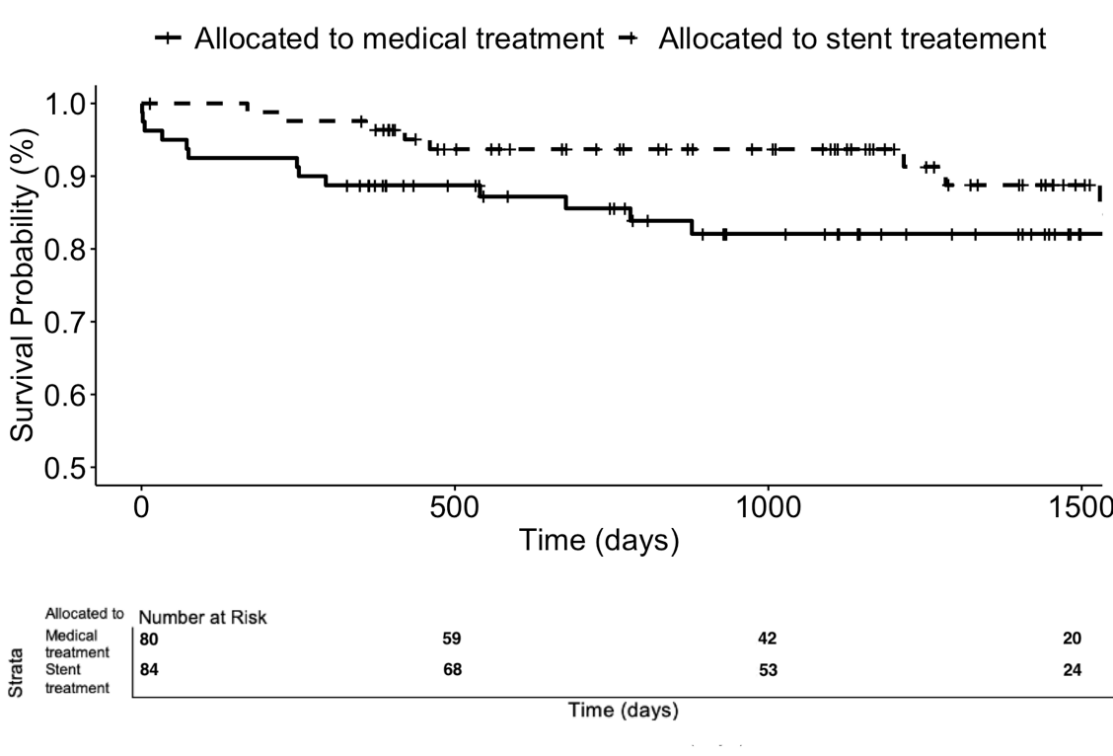


Supplementary Figure 3: Vertebrobasilar arterial distribution ischemic stroke free survival for patients who were randomized ≤ 30 days after a non-stroke qualifying ischemic event. (N=50) (adjusted HR: 1.600, 95% CI: 0.267–9.591, p=0.607)


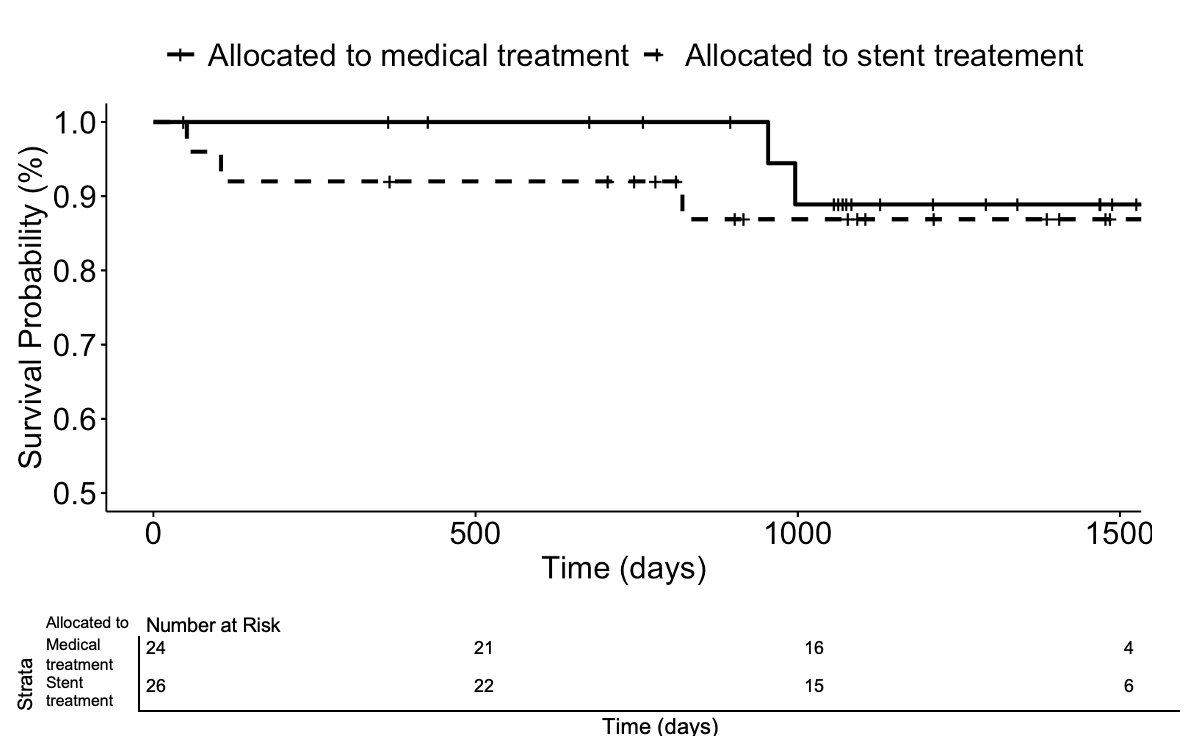


Stent group (dashed line) Medical group (solid line)

Supplementary Figure 4: Vertebrobasilar arterial distribution ischemic stroke free survival for patients who were randomized > 30 days after a non-stroke qualifying ischemic event. (N=30) (adjusted HR: ~0, 95% CI: 0–Inf, p=1.000)


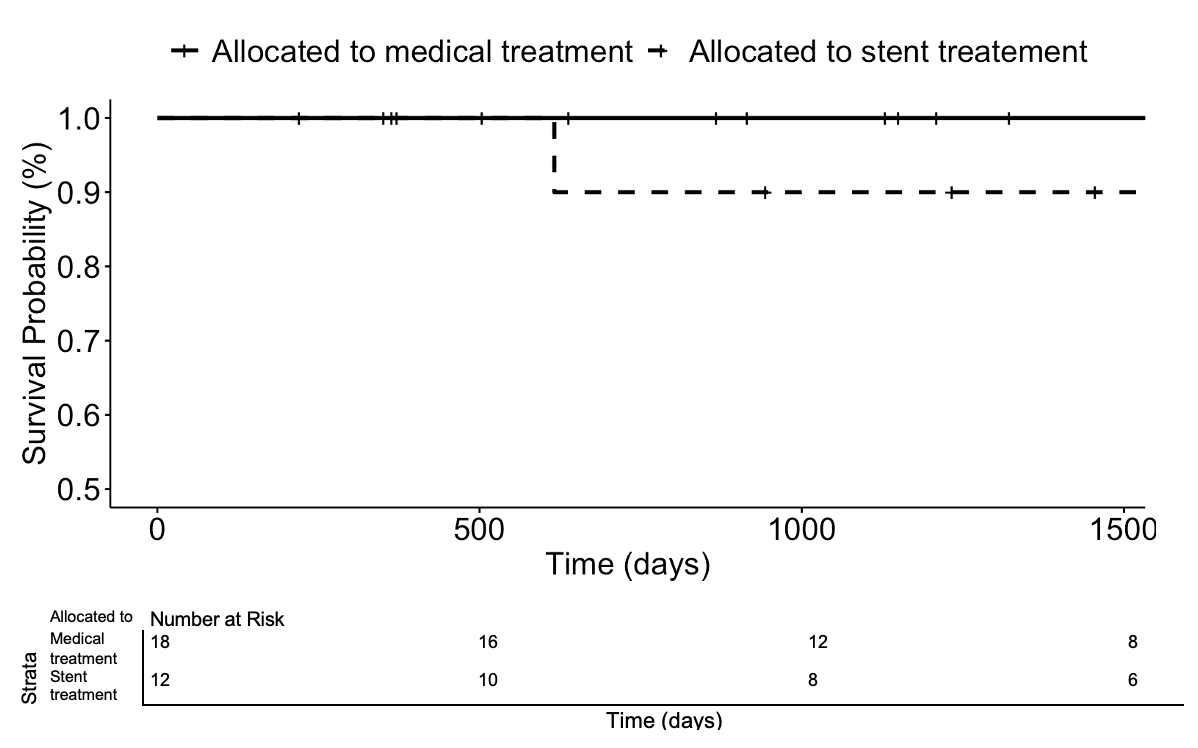


Stent group (dashed line) Medical group (solid line)

Supplementary Figure 5: Vertebrobasilar arterial distribution ischemic stroke free survival for patients who were randomized within any number of days after a non-stroke qualifying ischemic event. (N=80) (adjusted HR: 2.402, 95% CI: 0.440–13.120, p=0.312)

Stent group (dashed line) Medical group (solid line)


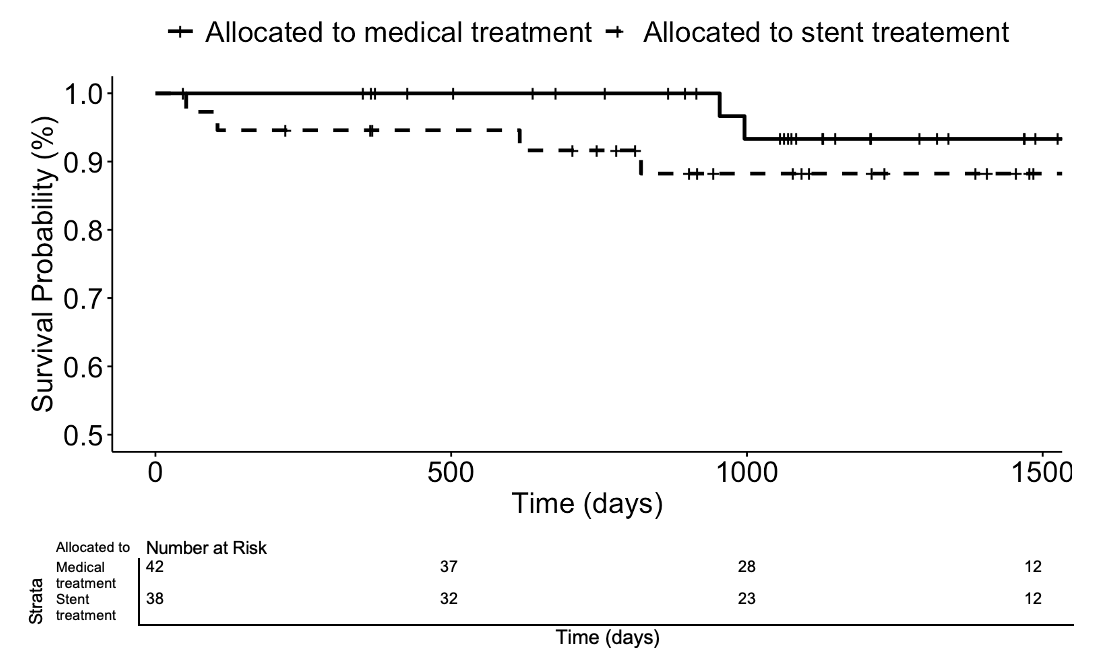


Supplementary Figure 6: Vertebrobasilar arterial distribution ischemic stroke free survival for patients who were randomized ≤ 30 days after any qualifying ischemic event. (N=144) (adjusted HR: 0.620, 95% CI: 0.249–1.542, p=0.304)

Stent group (dashed line) Medical group (solid line)


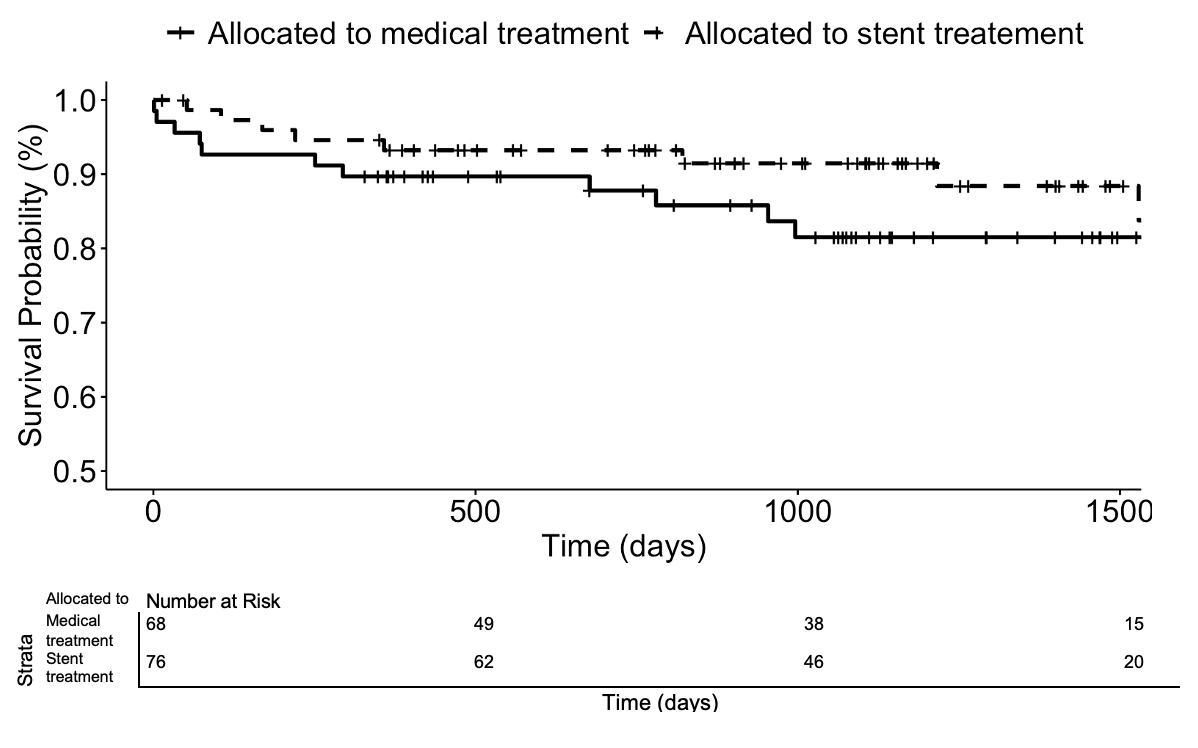


Supplementary Figure 7: Vertebrobasilar arterial distribution ischemic stroke free survival for patients who were randomized > 30 days after any qualifying ischemic event. (N=100) (adjusted HR: 1.160, 95% CI: 0.290–4.643, p=0.833)

Stent group (dashed line) Medical group (solid line)


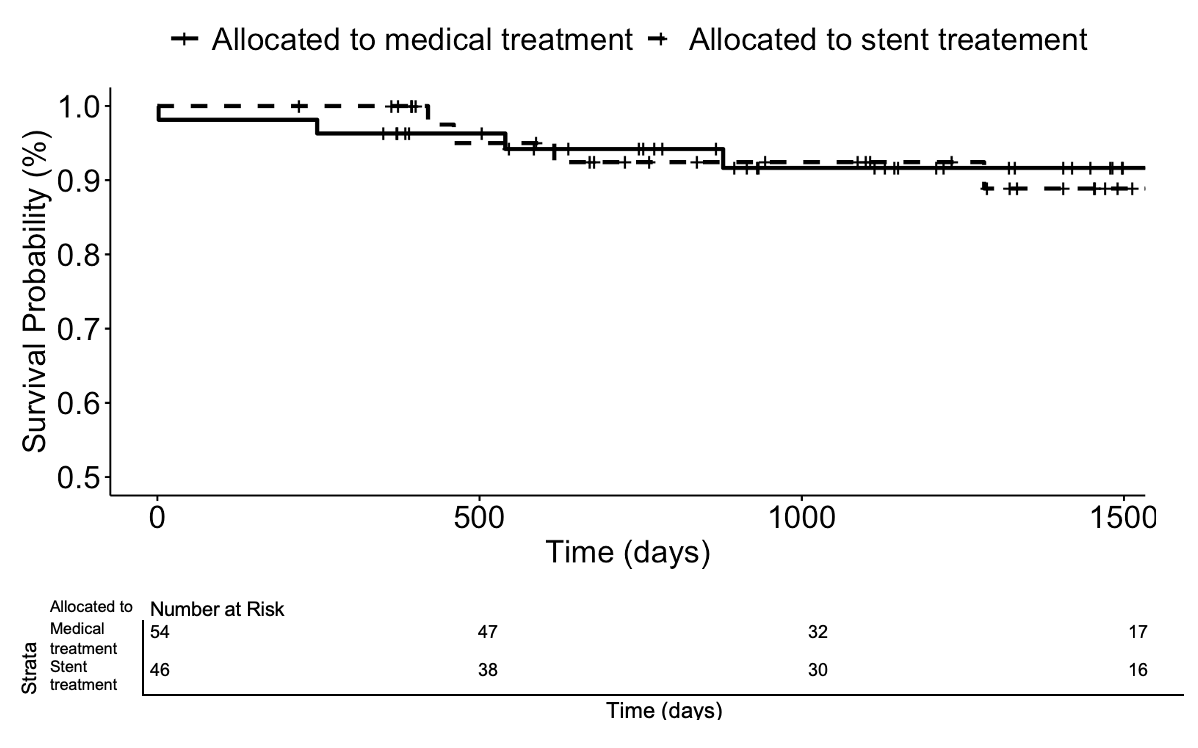


Supplementary Figure 8: Vertebrobasilar arterial distribution ischemic stroke free survival for patients who were randomized within any number of days after any qualifying ischemic event (Overall cohort). (N=244) (adjusted HR: 0.772, 95% CI: 0.361–1.649, p=0.504)


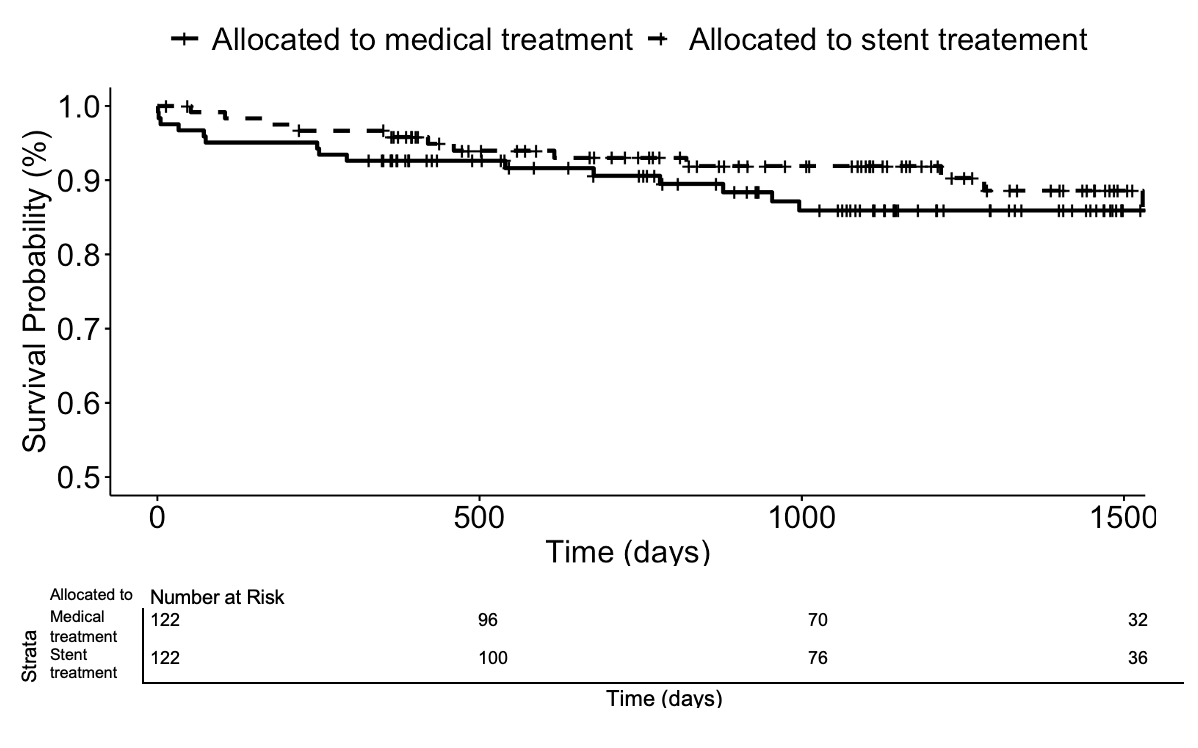


Stent group (dashed line) Medical group (solid line)

Supplementary Figure 9: CONSORT Flow Diagram: Selection and Stratification of Vertebral Artery Disease Patients in the first cohort

**
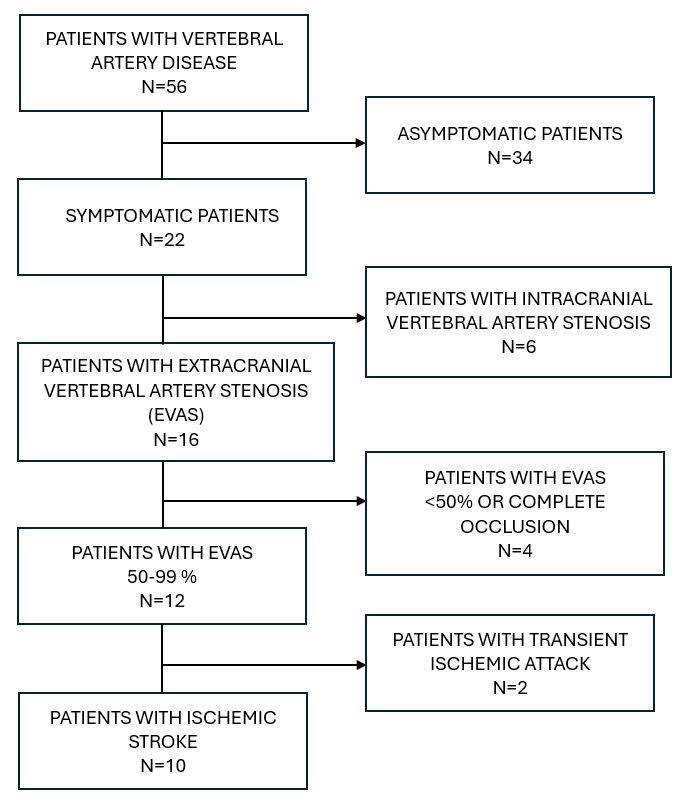
**

Abbreviations used: CONSORT, Consolidated Standards of Reporting Trails; EVAS, Extracranial Vertebral Artery Stenosis

Supplementary Figure 10: CONSORT Flow Diagram: Selection and Stratification of Vertebral Artery Disease in Patients in the second cohort.


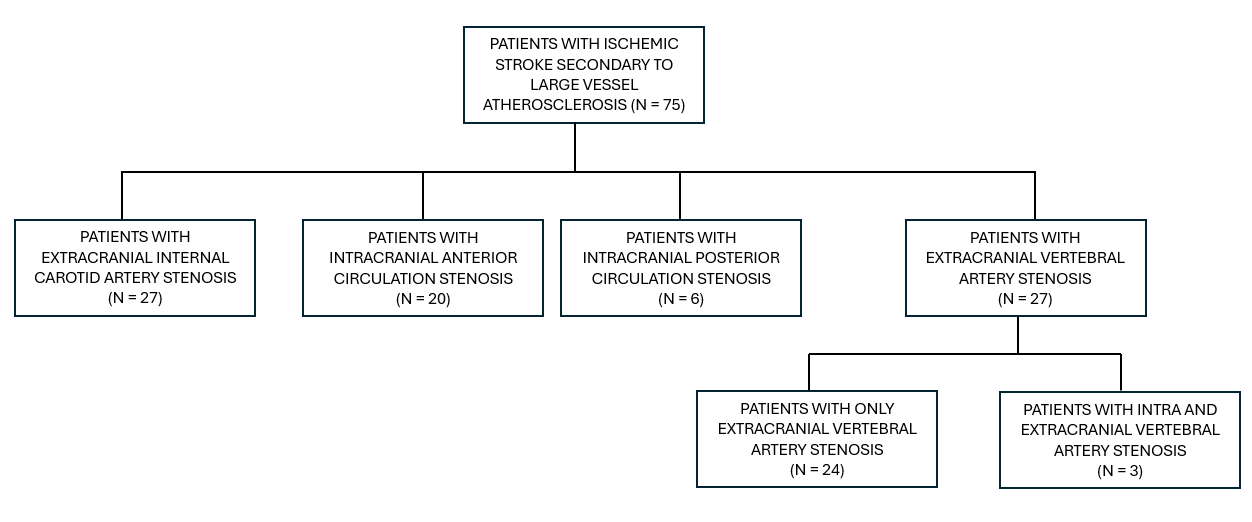


Abbreviations used: CONSORT, Consolidated Standards of Reporting Trails
